# Nanogene editing drug delivery systems in the treatment of liver fibrosis

**DOI:** 10.3389/fmed.2024.1418786

**Published:** 2024-09-25

**Authors:** Qun Wang, Siyu Jia, Zihan Wang, Hui Chen, Xinyi Jiang, Yan Li, Peng Ji

**Affiliations:** ^1^College of Pharmacy and Chemistry & Chemical Engineering, Taizhou University, Taizhou, China; ^2^Department of International Medicine, The Second Hospital of Dalian Medical University, Dalian, China; ^3^Liangzhu Laboratory, Zhejiang University School of Medicine, Hangzhou, China

**Keywords:** gene editing, delivery system, liver fibrosis, CRISPR-Cas9, nanoparticles, targeted therapy

## Abstract

Liver fibrosis is a group of diseases that seriously affect the health of the world’s population. Despite significant progress in understanding the mechanisms of liver fibrogenesis, the technologies and drugs used to treat liver fibrosis have limited efficacy. As a revolutionary genetic tool, gene editing technology brings new hope for treating liver fibrosis. Combining nano-delivery systems with gene editing tools to achieve precise delivery and efficient expression of gene editing tools that can be used to treat liver fibrosis has become a rapidly developing field. This review provides a comprehensive overview of the principles and methods of gene editing technology and commonly used gene editing targets for liver fibrosis. We also discuss recent advances in common gene editing delivery vehicles and nano-delivery formulations in liver fibrosis research. Although gene editing technology has potential advantages in liver fibrosis, it still faces some challenges regarding delivery efficiency, specificity, and safety. Future studies need to address these issues further to explore the potential and application of liver fibrosis technologies in treating liver fibrosis.

## 1 Introduction

Liver fibrosis is a chronic process disease caused by a variety of etiological factors, including alcoholism, chronic viral hepatitis, alcoholic hepatitis, non-alcoholic steatohepatitis (NASH), primary biliary cirrhosis, autoimmune diseases, and metabolic diseases (1, 2). The disease is mainly characterized by excessive and abnormal deposition of extracellular matrix (ECM), which is a self-repair mechanism of the liver in response to chronic injury caused by various aetiological factors, and hepatic fibrosis ultimately progresses to advanced liver diseases such as cirrhosis and hepatocellular carcinoma (3, 4). Currently, anti-fibrotic drugs under investigation mainly include herbal extracts (e.g., flavonoids), peroxisome proliferator-activated receptor-gamma (PPARy) agonists, tyrosine kinase inhibitors, hedgehog signaling inhibitors, bile acids, etc. (3, 5). Still, no biological or chemical anti-fibrotic drugs are approved for clinical use, so there is an urgent need for new and effective therapeutic agents in this field.

Liver fibrosis is reversible in the early stages of acute or self-limiting injury, and the liver can return to normal (1, 4). Still, in the case of persistent injury, chronic inflammation and ECM accumulation will persist, eventually leading to cirrhosis (6). Hepatic stellate cells (HSCs) and koilocytes (KCs) are the major ECM-producing cells and play a key role in the progression of liver fibrosis (7). HSCs and KCs have become the main targets for drug delivery to fibrotic regions using nanotechnology, and extensive basic experimental studies have been conducted on the role and potential applications of several pro-fibrotic genes expressed by HSCs and KCs in the progression of liver fibrosis (8). Effective gene editing methods to correct these pathological alterations at the gene level are expected to reverse the pathological process of liver fibrosis and achieve the therapeutic goal of liver fibrosis.

Gene editing is a genetic engineering technique that involves inserting, deleting, modifying, or replacing DNA sequences at specific locations within an organism’s genome (9). Common gene editing techniques include the following three main types: zinc finger nucleases (ZFNs), transcription activator-like effector nucleases (TALENs), clustered regularly interspaced short palindromic repeats, and TALENs, clustered regularly interspaced short palindromic repeats and CRISPR-associated proteins (CRISPR/Cas). These three gene-editing technologies have made important breakthroughs in disease treatment research, and the CRISPR/Cas system, in particular, has been widely used as a revolutionary gene-editing tool in disease research.

Gene editing relies on the delivery of a single guide (sgRNA) to a predetermined site and the targeted cleavage of DNA by Cas endonuclease, which can lead to genotoxicity due to DNA double-strand breaks (DSBs) at the target or off-target genes (10); therefore, ideal gene editing requires effective delivery tools to limit unintended editing of the genome (11). Nanoparticle vector-mediated gene editing delivery systems allow specific targeting of gene editing tools to intrahepatic fibrotic regions and intrahepatic cells for anti-hepatic fibrosis effects (12). This article provides a comprehensive overview of the principles and methods of gene editing technology and commonly used gene editing targets for liver fibrosis and discusses recent advances in common gene editing delivery vectors and nanoparticle delivery formulations in liver fibrosis research. Although gene editing technology has potentially great advantages in the field of liver fibrosis, it still faces some challenges in terms of delivery efficiency, specificity, and safety. Future studies need to address these issues further to explore in depth the potential and application of liver fibrosis technologies in the treatment of liver fibrosis.

## 2 Pathophysiology of liver fibrosis

**Figure 1:**
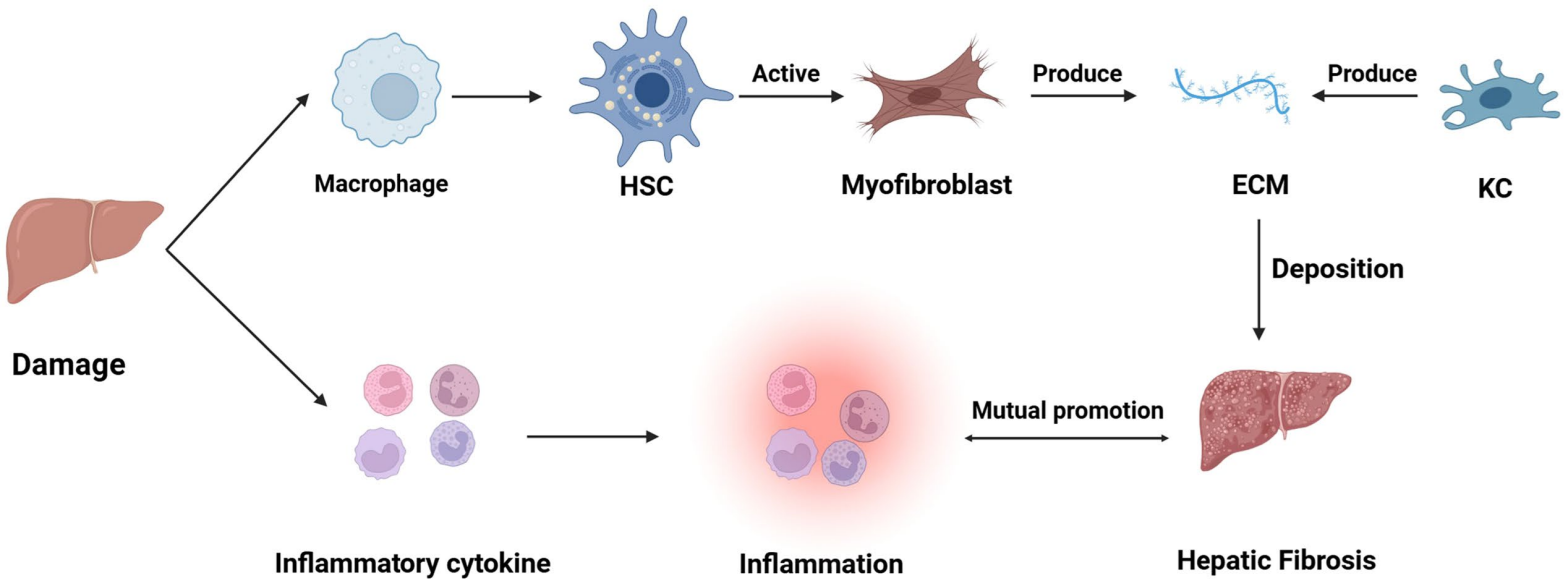
The mechanism of liver fibrosis.

HSCs are located in the Disse space, and after chronic injury, HSCs activate or differentiate into myofibroblasts; activated HSCs migrate and accumulate at sites of tissue repair, leading to an increase in their proliferation and contraction as well as the release of pro-inflammatory, pro-fibrotic and pro-mitotic cytokines, which secrete large amounts of ECM that regulate ECM degradation (13). In the normal liver, ECM is a highly dynamic matrix capable of controlling a precise balance between synthesis and degradation. However, during chronic liver injury, ECM production exceeds ECM degradation, leading to thickening of the fibrotic septa and chemical cross-linking of collagen, resulting in liver fibrosis. In addition, these ECM changes May also directly stimulate fibrogenesis (14). Hepatic fibrosis results from the liver’s wound-healing response to repeated injury, a process associated with an inflammatory response and limited deposition of ECM (15). In addition to HSCs, other hepatocyte types May potentially contribute to the development of fibrosis. For example, in cholestasis-induced liver fibrosis, myofibroblasts from small portal vessels proliferate around the bile ducts, initiating collagen deposition (16–18). After the liver injury, cells such as KCs, hepatocytes, HSCs, natural killer (NK) cells, lymphocytes, and dendritic cells also secrete cytokines, which can lead to a vicious cycle of mutual stimulation of inflammatory and fibrotic cells (e.g., Figure 1) (19). In addition, koilocytes, resident macrophages, also play an important role in liver inflammation by releasing ROS and cytokines (20).

In recent years, important progress has been made in understanding the pathological mechanisms of liver fibrosis. For example, FUNDC1 was found to bind directly to mitochondria and recruit GPx4. When mitochondria are severely damaged, mitochondrial autophagy is activated, and GPx4 that enters the mitochondria is degraded along with the damaged mitochondria, triggering iron death and exacerbating liver injury due to liver fibrosis. Liu et al. showed that hepatocyte exosome-derived MASP1 activates hematopoietic stem cells and promotes liver fibrosis, providing a new direction in the search for novel therapeutic targets (11). In addition, although inducing apoptosis in hematopoietic stem cells is a therapeutic strategy, the inactivation of hematopoietic stem cells or myofibroblasts May be a potential new target for reversing liver fibrosis, as inactivated hematopoietic stem cells May be more easily reactivated than before inactivation. Finally, liver fibrosis May also be influenced by factors in other organs (e.g., intestine, muscle, and adipose tissue). Although liver fibrosis is usually asymptomatic in its early stages, progression to cirrhosis is associated with a significant increase in morbidity and mortality. Once cirrhosis develops, the natural history of the disease usually progresses from the compensated to the decompensated phase, which manifests as portal hypertension and liver failure. Portal hypertension is, therefore, a major complication of cirrhosis and can lead to death or the need for liver transplantation.

## 3 Overview of gene editing technology

Gene editing is a cutting-edge technology that allows precise modification of an organism’s genome and has made remarkable progress in several fields. Gene editing can add, replace, delete, and modify genes, enabling precise genome manipulation. In recent years, research into genome editing technology has deepened, and various innovative technologies have been developed, such as CRISPR-Cas9, zinc finger nucleases (ZFNs), and transcription activator-like effector nucleases (TALENs). Each of these technologies has the advantage of using specific endonucleases to cut double DNA strands at predetermined sites, greatly improving gene editing targeting (see Table 1).

**Table 1:** Genes associated with treatment of liver fibrosis.

Gene editing technology	Advantages	Disadvantages	Applications
CRISPR-Cas9	It does not require protein engineering to modify the target, has high specificity, can achieve gene editing of many cells quickly, can be applied to various organisms, is simple to use, more efficient, and relatively inexpensive.	There are also off-target effects that cause deletion, translocation, and breakage of large fragments of genomic DNA. In addition, the Cas9 protein can trigger an immune response during *in vivo* experiments, reducing gene editing efficiency.	Treating single-gene disorders such as sickle cell anemia and cystic fibrosis and correcting a genetic disease in mice that causes cataracts (22).
ZFN	Zinc finger proteins binding to the target DNA sequence can be precisely engineered to initiate the natural DNA repair process and induce site-specific recombination. This is the more mature of the three gene editing technologies.	Some small genes and genes with high homology may not be able to be knocked out effectively, large fragment genes are difficult to knock in with technology, the assembly process is not modular, the synthesis time is long, the assembly is difficult, and the cost is high.	Human clinical trials for the related genetic diseases Duchenne muscular dystrophy (DMD), hemophilia, and HIV treatment (87).
TALEN	TALENs have higher specificity than ZFNs and can efficiently edit and modify the genome. In addition, the DNA binding of TALENs is modular. It can be easily combined and modified to adapt to different target DNA sequences and flexibly applied to various organisms.	Modular design is difficult and requires specific expertise and skills. In addition, TALENs have a certain cytotoxicity and a negative effect on cell growth and division.	Phase 1/2a clinical trial for the treatment of relapsed or refractory non-Hodgkin lymphoma (r/r NHL) and potential treatment for HPV infection and cervical cancer (88).

### 3.1 CRISPR-Cas9 technology

**Figure 2:**
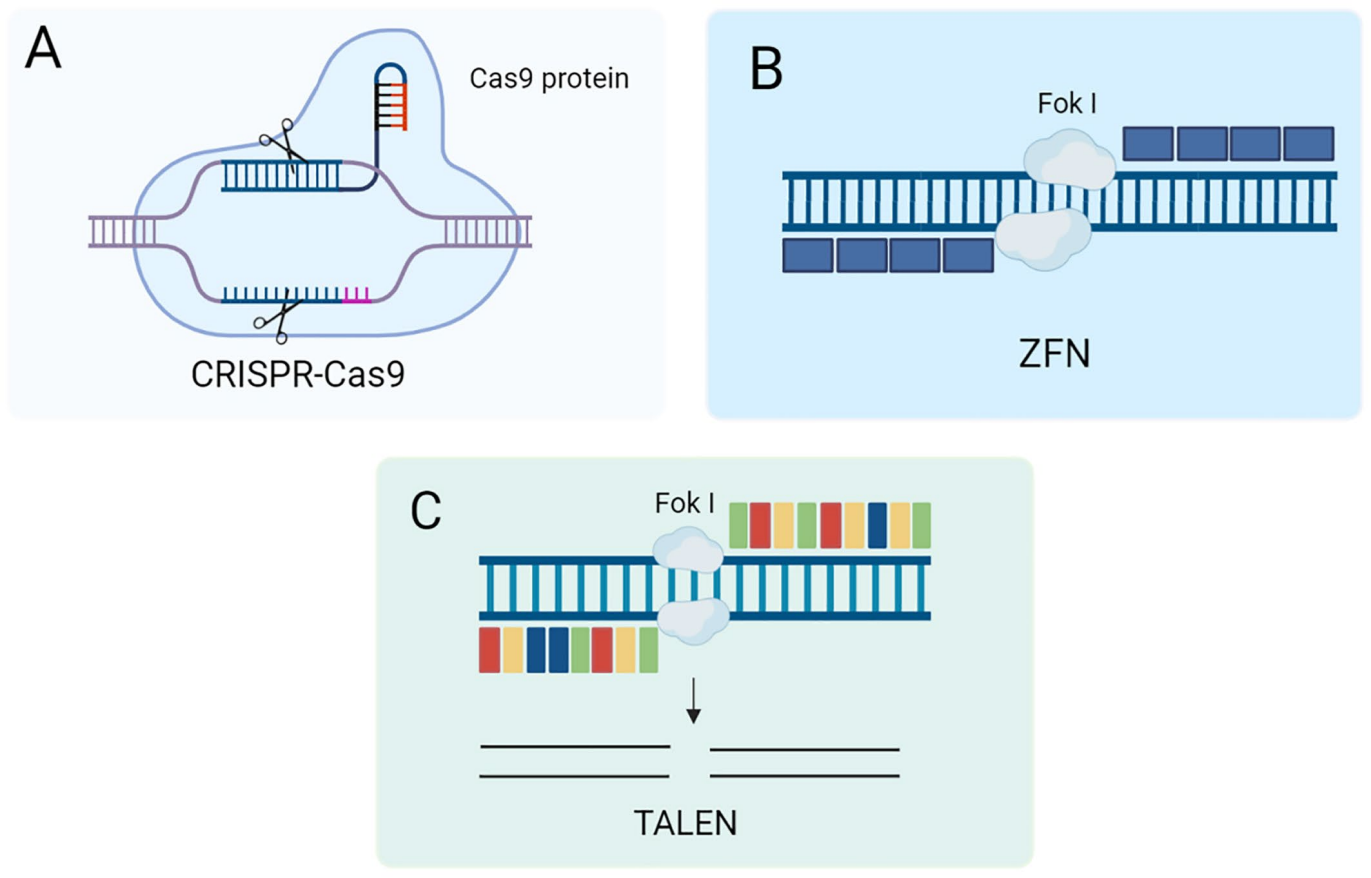
**(A)** Schematic of the CRISPR-Cas9 gene editing mechanism. **(B)** Schematic diagram of ZFN gene editing mechanism. **(C)** Schematic diagram of TALEN mechanism.

The CRISPR-Cas system is a remarkably adaptive and heritable acquired immune system found in bacteria and archaea that can integrate short sequences from viruses and other mobile genetic elements into host CRISPR genes (21). Among them, the CRISPR-Cas9 system is a family of DNA-specific targeting systems that can recognize target gene sequences by artificially designing sgRNAs to guide Cas9 proteases for efficient cleavage (e.g., Figure 2A) and is widely used for gene knockdown, gene silencing, and functional genome screening. In terms of delivery, the Cas9 ribonucleoprotein (RNP) has a large size, so its delivery vector has a higher loading capacity than other delivery vectors and shows great therapeutic potential in mouse models of acute liver injury, chronic liver fibrosis and hepatocellular carcinoma (22). As a biological frontier technology, gene editing with the CRISPR-Cas9 system holds great promise for treating liver fibrosis. CRISPR-Cas9 can precisely modify the key genes of liver fibrosis (such as ADAM9, GP96, Fas, etc.) to precisely and efficiently treat liver fibrosis and become a new approach for clinical treatment.

### 3.2 ZFN technology

ZFN technology is the first specific gene editing technology developed at the beginning of this century (23, 24). The technology is based on the fusion of two components to form the ZFN system. ZFN is a fusion of zinc finger proteins’ DNA binding domain with the FokI endonuclease cleavage domain (25). Zinc finger proteins play an important role in transcriptional regulation due to their specific DNA binding ability to recognize and bind specific DNA sequences (26). The FokI endonuclease is a restriction endonuclease with independent DNA recognition and cleavage functions (Figure 2B). By fusing these two protein structural domains, the ZFN system can achieve specific recognition and cleavage of DNA, thus showing a wide range of applications in biological research and gene therapy.

### 3.3 TALEN technology

TALEN is a DNA-binding protein originally discovered in gram-negative plant pathogens (27). It is based on a highly repetitive sequence that promotes homologous recombination *in vivo*. Like ZFN, TALEN consists of two key structural domains: a transcription activator-like effector (TALE) DNA-binding domain at the N-terminus and a restriction endonuclease FokI catalytic domain at the C-terminus. By fusing the DNA-binding domain of TALE and the catalytic domain of the FokI endonuclease, TALENs with potent gene editing capabilities have been generated (as shown in Figure 2C) (28). This fusion gives TALENs a significant advantage in gene editing, making them an important tool in modern molecular biology and biotechnology. TALEN technology has been widely used in various gene editing studies due to its high specificity and flexibility. Studies have shown that amniotic membrane mesenchymal stem cells (AMM/I) edited with TALEN technology can effectively improve liver function and acute liver fibrosis, and the secreted IL-10 has anti-inflammatory and antifibrotic effects (29). Therefore, TALEN editing technology can integrate an antifibrotic gene expression system into AMM/I, making it easily accessible and free from ethical issues. In addition, AMM/I are effective in preventing the development of liver fibrosis by reducing pro-inflammatory responses. AMM/I based on TALEN gene editing May become a promising alternative therapy for treating liver fibrosis.

### 3.4 Other editing techniques

#### 3.4.1 Prime editing

Prime editing is a novel technology based on the CRISPR-Cas9 system that can mediate targeted insertions, deletions, and 12-base substitutions for precise gene editing without DNA double-strand breaks (DSBs) and donor DNA templates (30). There are two core components: the primer-editing guide RNA (pegRNA) and the fusion protein. The pegRNA is based on sgRNA with an additional RNA sequence at the 3′ end, which can be used as a primer binding site for reverse transcription (PBS) and as a reverse transcriptase template (RTT). The fusion protein is obtained by fusing nCas9 (H840A mutant, which cleaves only the PAM-containing target DNA strand) to the reverse transcriptase. First, PAM is recognized by the pegRNA and binds to the target DNA strand complementary to the guide RNA, the PAM-containing target DNA strand is cleaved, and the cleaved target DNA strand is complementary to and binds to the PBS sequence at the 3′ end of the pegRNA, and the reverse transcription reaction is initiated. At the end of the reaction, the edited sequence and the original sequence of the target DNA compete for binding to the other strand through complementary base pairing, and the unbound sequence is twisted and finally excised, allowing gene editing. Gene therapy is an important application of gene editing tools; using gene editing tools to repair disease-causing mutations directly holds the promise of curing human genetic diseases. Recent studies have shown that the La protein is a key regulator that affects the efficiency of PE and has led to the development of a new PE system, PE7 (31). Now that the first PE technology has been approved for clinical use, it is believed that PE May soon have great potential in clinical gene therapy research.

#### 3.4.2 Base editing

Base editing is a novel genome editing tool based on the CRISPR-Cas9 system, consisting of a DNA-modifying enzyme fused to a programmable DNA targeting moiety that enhances the specificity and editing efficiency of the CRISPR system (32, 33). It can introduce point mutations without DSBs and without the need for a donor DNA template, resulting in efficient and stable base conversion. However, base editing techniques are often limited by the requirement for PAM sequences. The main building blocks of BE are fusion proteins and sgRNAs, where fusion proteins combine Cas9 cleavage enzymes and base modifying enzymes such as cytosine deaminase and adenine deaminase. The cytosine base editing (CBE) converts C-G bases to T-A bases, and the adenine base editing (ABE) converts A-T bases to G-C bases (34). The combination of CBE and ABE can efficiently perform four base conversions (C → T, G → A, A → G, T → C) but not the other eight (C → A, C → G, G → C, G → T, A → C, A → T, T → A, T → G), as well as insertions and deletions of bases, which also leads to the possibility of base conversions of the same bases near the target bases, thus reducing the efficiency of specificity editing. Liu et al. found that CBE can be used as a plasmid to correct glycosylation disorders caused by mutations (35). Although most of the results of BE studies are encouraging, there are still some difficulties in transporting large proteins and treating human genetic diseases.

## 4 Potential therapeutic targets for liver fibrosis

Gene editing technology has become an important tool for biomedical research, where the selection of targets is crucial for the accuracy of the editing. Fas, a cell surface receptor, is a key mediator in liver fibrosis: activation of the Fas pathway induces apoptosis in hepatocytes, a hallmark of liver injury and fibrosis (36, 37). Fas-mediated apoptosis not only exacerbates liver inflammation and injury but also accelerates the progression of liver fibrosis (38). Therefore, targeting the Fas pathway is a potential therapeutic strategy to alleviate liver fibrosis and its complications. Xiaojie Xu et al. developed a bispecific editing system for targeted delivery and specific editing against the liver (39). After intravenous injection, the system uses bionic macrophage membranes to efficiently deliver coding DNA or mRNA editing plasmids to inflamed liver tissue via a polymeric carrier. Intrahepatic transcriptional activity driven by the P3 promoter ensures precise regulation of gene expression and reduces off-target effects. Suppression of Fas gene expression protected mice from acute liver injury. In contrast, direct ablation of the Fas gene prevented the development of chronic hepatitis and alleviated symptoms associated with liver fibrosis.

Proprotein convertase subunit 9/kexin (PCSK9) May play a role in the progression of liver fibrosis (40). Although PCSK9 is primarily associated with cholesterol metabolism and cardiovascular disease, its expression in the liver and impact on hepatic lipid metabolism and inflammation have also attracted attention (41). Dysregulated lipid metabolism and chronic inflammation are key factors in liver fibrosis, and PCSK9 is thought to regulate these processes (42). However, the exact mechanism of PCSK9’s effect on liver fibrosis must be studied to determine its role in liver fibrosis and its potential as a therapeutic target (43). In particular, the research team successfully delivered CRISPR-Cas9 tools in a tissue-specific manner to organs such as the lung, liver, and kidney in mice (44, 45). *In vivo* experiments showed that liver-targeted SORT-LNP could effectively deliver Cas9 mRNA and sgPCSK9, significantly reducing serum PCSK9 protein levels by up to 90% in a mouse model of hypercholesterolemia.

Gp96 (glucose-regulated protein 96, also known as HSP90b I) is involved in the progression of liver fibrosis by regulating inflammatory responses, apoptosis, and the cell cycle (46). In a mouse model of alcoholic liver fibrosis, inhibition of Gp96 expression exacerbated liver fibrosis, suggesting that Gp96 plays a key role in liver protection (47). Knockdown of the gp96 gene using the CRISPR-Cas9 system showed that tail vein injection of Gp96-sgRNA3 expression plasmid significantly inhibited hepatic Gp96 expression, exacerbating alcoholic liver fibrosis. This suggests that Gp96 is protective in liver fibrosis (48). The human gp96 gene is located on chromosome 12, encodes 803 amino acids, and contains the signal peptide, the glycosylation site, and the endoplasmic reticulum retention sequence KDEL (49, 50). Silencing or modifying the gp96 gene using CRISPR-Cas9 is expected to lead to the development of gene therapy strategies targeting gp96 to inhibit liver fibrosis. Although CRISPR technology offers new insights and therapeutic avenues, its clinical translation is still challenging, and further research is needed to realize its therapeutic potential fully.

## 5 Commonly used delivery vectors for gene editing and their characteristics

Advancements in science and technology have increasingly highlighted the potential of gene editing for treating a wide range of diseases. However, the clinical implementation of these technologies is significantly challenged by the absence of safe and efficient delivery vectors. Thus, the development of effective and reliable delivery systems is imperative for the therapeutic application of gene editing. Delivery vectors for gene editing tools are primarily classified into viral and non-viral categories. Viral vectors mainly comprise adenoviruses and adeno-associated viruses, while non-viral vectors include synthetic carriers such as lipid nanoparticles.

### 5.1 Viral vectors

Adenovirus (AdV) is an envelope-less, double-stranded DNA that can cause symptoms of the common cold, conjunctivitis, and tonsillitis (51). AdV has a large loading capacity and can carry gene editing tools such as zinc finger nuclease (ZFN), transcription activator-like effector nuclease (TALEN), and clustered regularly spaced short palindromic repeats and their associated proteins (CRISPER/Cas) (52). In addition, adenoviruses have a high transfection rate, can infect a wide range of cell types, and have low genotoxicity *in vivo* (53). AdV is widely used in clinical therapy due to its wide range of infectious hosts, ease of purification, good genetic stability, and high exogenous gene load. However, due to its high immunogenicity and other problems, AdV still faces many challenges in clinical applications (54). Adeno-associated virus (AAV) is a non-pathogenic enveloped virus with single-stranded DNA (55). As the most popular viral vector, AAV is widely used in clinical practice due to its low immunogenicity, low genotoxicity, coat protein diversity, long duration of action, and high biocompatibility (56). AAV can efficiently deliver gene editing tools to target cells, and prime editing is a highly versatile CRISPR-based genome editing technology that works without causing DNA double-strand breaks. Böck et al. developed a reduced-size SpCas9 primary editor (PE) for adeno-associated virus-mediated delivery to the liver. They performed 14% primary editing at the Dnmt1 test site in mouse liver using a dual AAV8 vector (57). Efforts to recognize smaller immediate homologs of Cas9 or generate small engineered Cas9 variants have enabled single AAV delivery of CRISPR gene editors, and Zhang and colleagues used a single AAV vector encoding the SaCas9 nuclease to knock down Pcsk9 and reduce serum cholesterol in mice (58). Overall, the expression of gene editing tools that control AAV delivery provides a useful strategy to maximize gene editing specificity, potentially improving future therapeutic applications’ safety.

### 5.2 Non-viral vectors

Compared to viral vectors, non-viral vectors have the advantages of better biocompatibility, biodegradability, higher loading capacity, low cost, and ease of production (59). Due to their low toxicity and low immunogenicity, non-viral vectors are expected to replace viral vectors. Lipid nanoparticles (LNPs), a widely used therapeutic nucleic acid delivery vehicle, are typically tens to hundreds of nanometres and can penetrate cell membranes and deliver drugs into cells (60). LNP formulations currently approved for clinical use contain four lipid types: ionizable cationic lipids, phospholipids, cholesterol or cholesterol derivatives, and polyethylene glycol (PEG) lipids (61). Ionizable cationic lipids form complexes with negatively charged RNA molecules, and their pH sensitivity enhances the biocompatibility of LNP. At normal physiological pH, ionizable cationic lipids are uncharged, reducing their interaction with the anionic membranes of non-target cells. LNP preparations are usually carried out at acidic pH, where ionizable cationic lipids are protonated and able to bind negatively charged cargoes, destabilizing endosomal membranes and promoting the escape of RNA molecules (62). Therefore, incorporating ionizable cationic lipids into LNP formulations allows efficient payload encapsulation and improved *in vivo* circulation time and cellular uptake. Phospholipids are mainly found in the outer lipid layer of LNPs and play a role in stabilizing the LNP structure during particle formation. Combinations of phospholipids can be added to LNP formulations to modify their biophysical properties and promote optimal encapsulation, stability, and endosomal release (63). Cholesterol or its derivatives stabilize particles by modulating membrane integrity and rigidity, influencing particle distribution efficiency and biodistribution. PEG lipids enhance LNP stability, modulate particle size, prevent particle aggregation, reduce immunogenicity, and increase circulation time (64). These properties give lipid nanoparticles excellent biocompatibility and biodegradability, which protects biomolecules such as mRNA from degradation and ensures that these molecules reach the target cells intact, allowing lipid nanoparticles to be targeted for more precise delivery of drugs or vaccines to target tissues or cells (65). Extracellular vesicles, a general term for exosomes, microvesicles, and apoptotic vesicles, can encapsulate various molecules and transport them from the donor cell to the recipient cell, thereby altering the physiological function of the recipient cell. As endogenous vesicles, extracellular vesicles have low immunogenicity and are safe to use as gene therapy vectors (66). Ideal gene editing tool vectors should have targeting, stability, ease of preparation, low toxicity, and the ability for efficient gene transfer and long-term expression (67). As more *in vivo* gene editing therapies enter the clinical phase, reliable *in vivo* delivery methods will become critical. Advances in delivery technology will facilitate the realization of a wide range of *in vivo* gene editing therapies.

## 6 Gene editing delivery system for treatment of liver fibrosis

The development of drug delivery technologies has provided new ideas to improve the efficacy of anti-hepatic fibrosis and reduce toxic side effects. Currently, novel gene-edited drug delivery systems for liver fibrosis include lipid nanoparticles, micelles, exosomes, and gold nanorods (see Table 2). By designing delivery systems based on drug properties, it is possible to improve their solubility, achieve slow release, and increase bioavailability; in addition, improving the formulation and process of nanoparticles to adjust their surface properties or modify specific targeting molecules can help to achieve precise modulation and treatment of liver targets.

**Table 2:** Gene editing drug delivery systems for liver fibrosis.

Delivery vehicle	Drug	Mechanism of action	Design
Lipid Nanoparticles	Relaxin gene and miR-30a-5p mimics	Activation of hepatic stellate cells	Encapsulation of relaxin gene and miR-30a-5p mimics with lipid nanoparticles for synergistic treatment of hepatic fibrosis by combination gene therapy (71)
	Cas9 mRNA	Knockout of Angptl3 gene	Use LNP technology to package CRISPR-Cas9-mRNA and deliver it into mouse liver (73)
Polymer Nanoparticles	CRISPR/dCas9-miR-524	Upregulation of miR-524 and inhibition of Wnt-*β* protein and other signaling pathways	MDNPs are designed as core-shell structures to deliver payloads to tumor tissues (76)
	PD/P	Inducing Cas9 or CasRx expression	A biomimetic macrophage membrane is coated on the surface of the PD/P nano complex to deliver the plasmid to the inflammatory lesions of the liver (39)
Exosome carriers	Cas9 RNP	Regulate the expression of PUMA, CcnE1, and KAT5	Load Cas9 RNP into exosomes derived from liver fibroblasts outside the cell (ExosomeRNP) (79)
	CRISPR*/*dCas9-SAM	Activating HNF4α/HGF1/FOXA2 genes to improve CCL4-induced liver fibrosis	RBP4-modified exosomes deliver the CRISPR/dCas9 system to hematopoietic stem cells (80)
	CRISPR/dCas9-VP64	Activating the HNF4α transcriptional regulator significantly attenuates liver fibrosis	AML12 cell-derived exosomes loaded with CRISPR/dCas9-VP64 system are delivered to hematopoietic stem cells (81)
Gold Nanorods	CRISPR/Cas9	Cationic AuNRs were used to deliver Fas-targeting CRISPR/Cas9 plasmids *in vivo*, successfully protecting mice from liver fibrosis	Cationic polymer-coated AuNRs for CRISPR/Cas9 plasmid delivery (89)

### 6.1 Lipid nanoparticles

LNPs comprise lipid bilayers forming phospholipid vesicles with excellent biocompatibility, targeting, efficacy, and degradability (68). They enhance drug absorption and bioavailability, cross the blood–brain barrier, and prolong circulation time *in vivo* (69). Relaxin is an antifibrotic peptide hormone that reverses hepatic stellate cell activation and alleviates liver fibrosis (70). However, relaxin gene therapy could not restore activated hepatic stellate cells to a quiescent state *in vitro*. To address this issue, Hu et al. used lipid nanoparticles to encapsulate the relaxin gene and miR-30a-5p mimics and achieved a synergistic antifibrotic effect through combinatorial gene therapy in a mouse model of liver fibrosis, successfully targeting and treating activated hepatic stellate cells (71).

The development of CRISPR gene editing technology has had a significant impact on humans. It is now widely used in gene editing, gene therapy, nucleic acid localization, and detection to treat diseases by targeting specific genes. However, at the individual level, the CRISPR-Cas9 system is difficult to deliver efficiently to the patient site, resulting in low editing efficiency (72). To overcome this problem, Qiu et al. developed a lipid nanoparticle delivery system carrying Cas9 messenger RNA (mRNA) and guide RNA for CRISPR-Cas9-based Angptl3 genome editing *in vivo* (73). CRISPR-Cas9 mRNA encapsulated by LNP technology and delivered to the mouse liver deleted the gene called Angptl3, resulting in a 57% reduction in blood cholesterol levels in mice, and the effect of a single injection lasted for several months.

### 6.2 Polymer nanoparticles

Polymeric nanoparticles made from biodegradable polymers such as PLGA (poly(lactic acid)-hydroxyacetic acid) are used as drug carriers for controlled release and targeted delivery of insoluble drugs with reduced toxicity and improved cellular uptake efficiency (74). The drug delivery process can be optimized by tuning the nanoparticles’ surface properties, size, and shape. They can be designed as responsive nanosystems to release drugs in specific biological environments (e.g., tumor microenvironments), thereby reducing damage to normal tissues and improving therapeutic efficacy (75). In addition, polymeric nanoparticles can be combined with targeting molecules to achieve active, targeted drug delivery, further enhancing therapeutic efficacy. Liu et al. developed a multistage delivery nanoparticle (MDNP) with a core-shell structure composed of responsive polymers, which enables the MDNP to adjust surface properties according to changes in the surrounding microenvironment and deliver the payload to tumor tissue with optimal efficiency (76). The nanoparticles enable tumor-targeted delivery of the CRISPR/dCas9 system and restore endogenous microRNA (miRNA) expression *in vivo*. The study showed that MDNP/dCas9-miR-524 effectively upregulated miR-524 in tumors in hormone-treated mice, interfered with multiple pathways associated with cancer cell proliferation, and significantly inhibited tumor growth, validating the therapeutic potential of MDNP in CRISPR/dCas9 tumor-targeted delivery. In addition, Xu et al. developed a dual liver-specific CRISPR editing nanosystem that uses bionic macrophage membranes to target plasmids carrying DNA or mRNA editors to inflammatory liver tissue and regulate their transcriptional activity in the liver via the P3 promoter. The system achieved efficient liver-specific editing in a mouse model of liver fibrosis. Disruption of the Fas gene can effectively halt the progression of chronic hepatitis and alleviate the symptoms of liver fibrosis (39).

### 6.3 Exosome carriers

Exosomes are nanovesicles naturally released by cells and possess biocompatibility, transport capacity, and blood flow stability (77). Exosomes have low immunogenicity and fewer toxic side effects than other non-viral vectors, making them ideal for delivering gene-edited ribonucleoprotein complexes (78). CRISPR-Cas9 technology has demonstrated unique advantages in disease treatment. The delivery of Cas9 ribonucleoprotein complexes (Cas9 RNPs) bypasses the intracellular transcription and translation processes required for Cas9 DNA/mRNA, reducing immunogenicity and off-target effects. However, many RNPs exceed the current delivery vectors’ loading capacity. Wan et al. successfully prepared exosome gene editing nanoparticles (ExosomeRNP) using an optimized electroporation method to load Cas9 RNP directly into liver fibroblast-derived exosomes to address this issue (79). The nanoparticles efficiently delivered RNP to target cells and produced significant gene editing effects. It was found that the exosome RNP showed strong therapeutic potential in mouse models of acute liver injury, chronic liver fibrosis, and hepatocellular carcinoma by targeting p53 to upregulate apoptosis regulator (PUMA), cell cycle protein E1 (CcnE1) and K (lysine) acetyltransferase 5 (KAT5). Luo et al. found that the CRISPR/dCas9-VP64 system could efficiently deliver to hepatic stellate cells (HSC) by encapsulating AML12 cell-derived exosomes. The CRISPR/dCas9-VP64 system has an efficient gene transcriptional activation function in which sgRNA recognizes and binds to complementary sequences of target genes and precisely directs dCas9-VP64 to target genes. As a key transcriptional regulator of hepatocyte differentiation, activation of the HNF4a gene significantly attenuates liver fibrosis (80). Therefore, effective activation of HNF4a gene transcription by the CRISPR/dCas9-VP64 system in HSC is expected to slow down the process of liver fibrosis. In addition, Luo et al. successfully delivered the CRISPR/dCas9-VP64 system to hepatic stellate cells (HSC) via exosomes derived from AML12 cells. CRISPR/dCas9-SAM plasmid DNA was encapsulated in exosomes, and RBP4 exosomes were isolated from AML12 cells expressing the CRISPR/dCas9-SAM lentiviral vector. These exosomes effectively reversed ccl4-induced liver fibrosis by activating HNF4α, HGF1, and FOXA2 genes after successful delivery to the liver (81). Despite the great potential of exosomes for encapsulated drug delivery, there is still much room for improvement in encapsulation efficiency and numerous challenges for *in vivo* therapy.

### 6.4 Gold nanorods

In recent years, gold nanomaterials have emerged as promising delivery vehicles for various biomedical applications (82, 83). Gold nanorods (AuNRs) are rod-shaped gold nanoparticles with lengths ranging from tens to hundreds of nanometres, unique nanostructures, excellent photothermal effects, and rich physicochemical properties. Studies have shown that knockdown of the Pcsk9 gene using hepatocyte-targeted gold cluster-based nanoparticles mediating CRISPR/Cas9 delivery *in vivo* reduces systemic LDL cholesterol levels, demonstrating its therapeutic potential in preventing cardiovascular disease. However, the transfection efficiency of these vectors is moderate, and effective delivery of Cas9 and sgRNA remains a challenge. Therefore, developing new materials capable of transfecting large CRISPR/Cas9 plasmids more efficiently is critical. Chen et al. designed a series of cationic polymer-coated gold nanorods (AuNRs) to deliver CRISPR/Cas9 plasmids. The results showed that the high aspect ratio (AR) cationic polymer-coated AuNRs exhibited unique DNA assembly, excellent internalization-mediated ability, and strong endosome escape. It was further demonstrated that such optimized high AR cationic AuNRs could effectively deliver CRISPR/Cas9 plasmids to various cell types. *In vivo*, delivery of CRISPR/Cas9 plasmids targeting Fas via cationic AuNRs successfully protected mice from liver fibrosis. Nanomaterials with high AR are currently emerging as drug delivery vehicles, and this discovery opens new avenues for developing advanced delivery materials for therapeutic genome editing.

## 7 Challenges and limitations

The liver is a structurally complex organ containing multiple cell types. Tools such as the CRISPR genome editing system, TALENs, and ZFNs are important in biomedical research and gene therapy. However, the recognition efficiency of nuclease enzymes such as Cas9 is affected by various factors and can trigger editing at non-target sites, posing a potential safety risk. Even if they are successfully delivered to the target cells, it is still necessary to ensure their efficient operation to avoid ineffective editing. In addition, gene editing tools, such as viral vectors, can trigger an immune response in the host, reducing editing efficiency and leading to adverse effects.

### 7.1 Off-target effects

Off-target effects are a major risk of gene editing, which can lead to unintended mutations, disrupt cellular functions, and even cause diseases such as cancer. Cellular environmental factors, such as DNA repair mechanisms and cell cycle phases, affect editing efficiency and off-target risks. Controlling off-target effects is critical because the CRISPR-Cas9 system can induce permanent genomic changes. Researchers have developed variants such as eSpCas9 and SpCas9-HF1 to reduce off-target effects, but in-depth studies are needed to improve editing fidelity (84).

### 7.2 Immune response

Nanocarriers and gene-editing components can induce an immune response in the host, affecting the safety and efficacy of gene-editing therapies (85). The immune response attenuates the therapeutic effect and poses a safety risk. The addition of immunosuppressive agents (e.g., corticosteroids) can mitigate this response. LNPs perform well in nucleic acid delivery, and polymeric materials are promising due to their longer blood circulation time and excellent biocompatibility. Reducing the immune response is a pressing issue in gene editing therapies.

### 7.3 Stability and toxicity

Nanocarriers’ stability and potential toxicity are important considerations for drug delivery systems. Although nanocarriers have advantages in improving drug bioavailability and targeting, they are associated with potential biosafety issues. Lipid-based nanomaterials such as LNPs have made important advances in drug delivery, but their toxicity is influenced by several factors, such as lipid composition and surface charge (86). Optimization of nanocarrier design and surface modification can reduce toxicity and improve therapeutic efficacy.

## 8 Summary and outlook

Liver fibrosis remains a significant global health challenge, with current therapeutic options offering limited efficacy. Integrating gene editing technologies, particularly CRISPR-Cas9, with advanced nano-delivery systems represents a promising approach to overcoming these challenges. This manuscript has reviewed the potential of various gene editing tools, such as CRISPR-Cas9, ZFNs, and TALENs, in targeting critical genes involved in liver fibrosis. It has also explored the efficacy of various nanocarriers, including lipid nanoparticles, polymeric nanoparticles, and exosome-based carriers, in improving gene editing therapies’ specificity, efficiency, and safety. While significant progress has been made, challenges such as off-target effects, immune responses, and delivery efficiency remain to be addressed.

Future research should focus on improving the precision and safety of gene editing technologies to minimize off-target effects and improve therapeutic outcomes. Developing more sophisticated and biocompatible nanocarriers will be crucial for the successful clinical translation of these therapies. In addition, interdisciplinary collaboration between researchers in gene editing, nanotechnology, and hepatology is essential to advance the field. Continued exploration of novel gene targets and refinement of delivery systems May pave the way for more effective and personalized treatments for liver fibrosis, ultimately improving patient outcomes and quality of life.
